# Deep Learning for Virtual Histological Staining of Bright-Field Microscopic Images of Unlabeled Carotid Artery Tissue

**DOI:** 10.1007/s11307-020-01508-6

**Published:** 2020-06-08

**Authors:** Dan Li, Hui Hui, Yingqian Zhang, Wei Tong, Feng Tian, Xin Yang, Jie Liu, Yundai Chen, Jie Tian

**Affiliations:** 1 Department of Biomedical Engineering, School of Computer and Information Technology, Beijing Jiaotong University, Beijing, 100044 China; 2 CAS Key Laboratory of Molecular Imaging, Institute of Automation, Chinese Academy of Sciences, Institute of Automation, Beijing, 100190 China; 3 University of Chinese Academy of Sciences, Beijing, 100190 China; 4 Department of Cardiology, Chinese PLA General Hospital, Beijing, 100853 China; 5 Beijing Advanced Innovation Center for Big Data-Based Precision Medicine, School of Medicine, Beihang University, Beijing, 100083 China

**Keywords:** Virtual histological staining, Conditional generative adversarial network, Blind evaluation, Bright-field microscopic imaging

## Abstract

**Purpose:**

Histological analysis of artery tissue samples is a widely used method for diagnosis and quantification of cardiovascular diseases. However, the variable and labor-intensive tissue staining procedures hinder efficient and informative histological image analysis.

**Procedures:**

In this study, we developed a deep learning-based method to transfer bright-field microscopic images of unlabeled tissue sections into equivalent bright-field images of histologically stained versions of the same samples. We trained a convolutional neural network to build maps between the unstained images and histologically stained images using a conditional generative adversarial network model.

**Results:**

The results of a blind evaluation by board-certified pathologists illustrate that the virtual staining and standard histological staining images of rat carotid artery tissue sections and those involving different types of stains showed no major differences. Quantification of virtual and histological H&E staining in carotid artery tissue sections showed that the relative errors of intima thickness, intima area, and media area were lower than 1.6 %, 5.6 %, and 12.7 %, respectively. The training time of deep learning network was 12.857 h with 1800 training patches and 200 epoches.

**Conclusions:**

This virtual staining method significantly mitigates the typically laborious and time-consuming histological staining procedures and could be augmented with other label-free microscopic imaging modalities.

**Electronic supplementary material:**

The online version of this article (10.1007/s11307-020-01508-6) contains supplementary material, which is available to authorized users.

## Introduction

Coronary artery disease (CAD) is the leading cause of mortality globally. Fundamental studies regarding CAD pathophysiological mechanisms and potential therapeutic methods have considerable clinical and scientific significance, which highly rely on histology analysis of artery tissue sections [1–4]. Histological staining can be used to identify arterial features such as intima, media, collagen, and elastic lamina. However, histological staining of artery tissue is an invasive and laborious process that typically includes the artery tissue being fixed and paraffin-embedded, sectioned into 2–10-μm thin slides, chemically stained or fluorescently labeled, mounted on a glass slide, and imaged using a bright-field microscope. Inevitably, the variability of histologically stained tissue sections in these irreversible steps introduces major challenges in histopathological image analysis. These variations are due to human-to-human variability, differences in protocols and microscopes between labs, and color variations in staining procedures [5]. More importantly, to identify various arterial features, multiple stains of the same tissue section are required, whereas the standard histological staining procedure is applied for one type of stain on single artery sections. The time-consuming histological staining procedures also create obstacles for fast pathological diagnosis. Recently, tissue-sectioning microscopies, such as confocal and multiphoton microscopes, have been applied for non-invasive volumetric or quantitative measurements of artery tissue sections to accelerate and improve the microscopic imaging step in this workflow [6, 7]. Nevertheless, tissue-sectioning microscopy requires fluorescence agents as imaging probes in contrast to specific artery tissue compositions.

The use of nonlinear microscopy has been suggested for visualization of unstained tissue samples based on tissue autofluorescence [8, 9], two-photon fluorescence [6, 10], second-harmonic generation [11], third-harmonic generation [12], and Raman scattering [13, 14]. Moreover, optoacoustic imaging has also been investigated for label-free imaging of red blood cells in atheroma [15, 16]. Furthermore, multimodal multiphoton and optoacoustic microscopy was proposed for hybrid imaging of histological features and moieties in excised human carotid atheroma [17]. However, these microscopy methods require ultrafast pulse lasers or spectrometers, which have lower output power, and their implementation on a microscope is a complex process. Therefore, they might not be readily available in most settings and require relatively long scanning times because of the weak optical signals.

Although the aforementioned state-of-the-art microscopy techniques have unique capabilities to visualize different histological moieties in tissue samples using exogenous staining, autofluorescence, or intrinsic information, pathologists are trained to examine histologically stained tissue samples to make diagnostic decisions. Inspired by this, recently, efforts have been focused on virtually creating histological staining images by training microscopic images of tissue samples *via* deep learning-based methods. Rivenson et al. trained a network that maps the autofluorescence microscopic images of unlabeled tissues to the histological staining images using a convolutional neural network (CNN) [18]. Another study used conditional generative adversarial networks (cGANs) to transfer unstained hyperspectral lung histological images to their corresponding hematoxylin and eosin (H&E) staining images [19]. To address the problem of stain color variations, as approach based on unpaired image-to-image translation using cycle-consistent adversarial networks has also been proposed [20]. Bautista and Yagi [21] proposed a method to transform H&E-stained multispectral images into its Masson’s trichromatic stained equivalent by enhancement spectral transmittance and linear transformation.

**Fig. 1:**
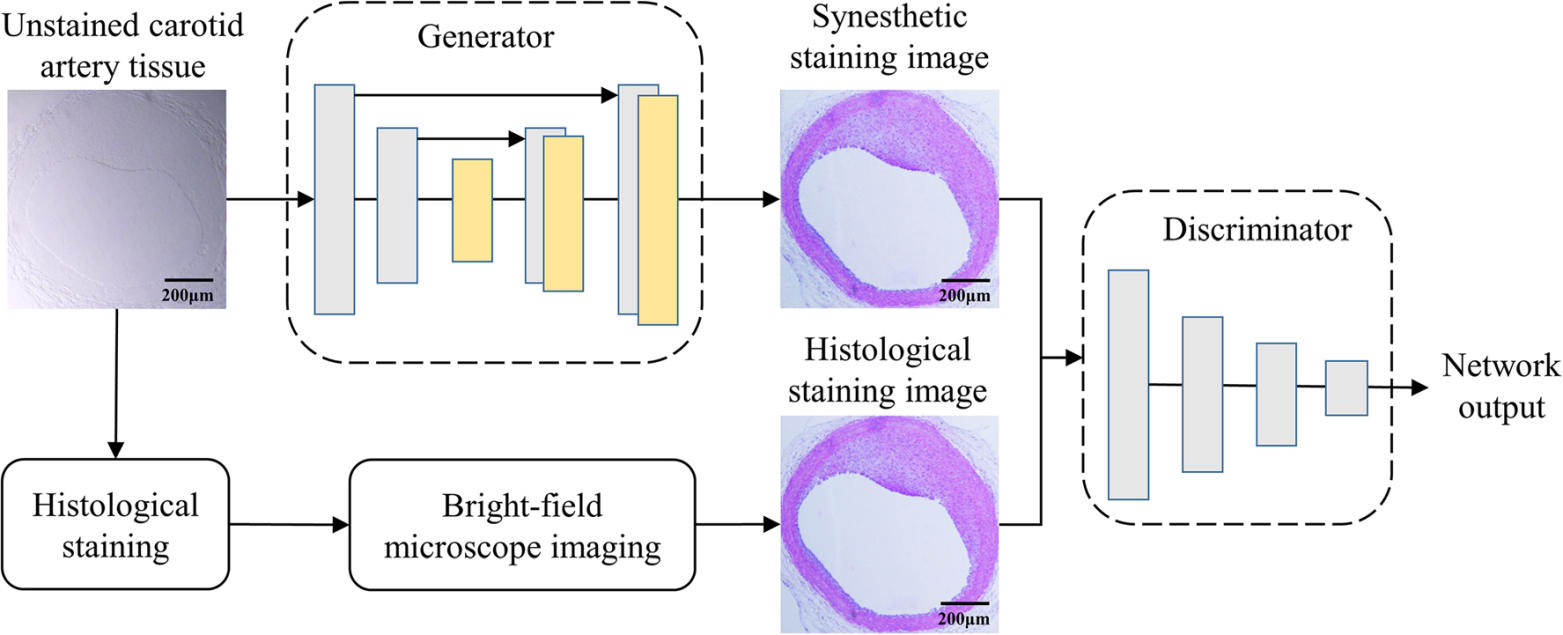
Main framework of the proposed virtual histology staining method of unstained carotid artery tissue using the conditional generative adversarial network. The bright-field images of unstained carotid artery cross-sections are fed into the generator network to generate synesthetic staining images (top). The standard histological staining (bottom) process is performed to output histological staining image. After discriminator network, the cGAN outputs a virtually stained image (H&E in this case) in response to the input of a bright-field image of an unstained tissue section, bypassing the standard histological staining procedure.

**Fig. 2:**
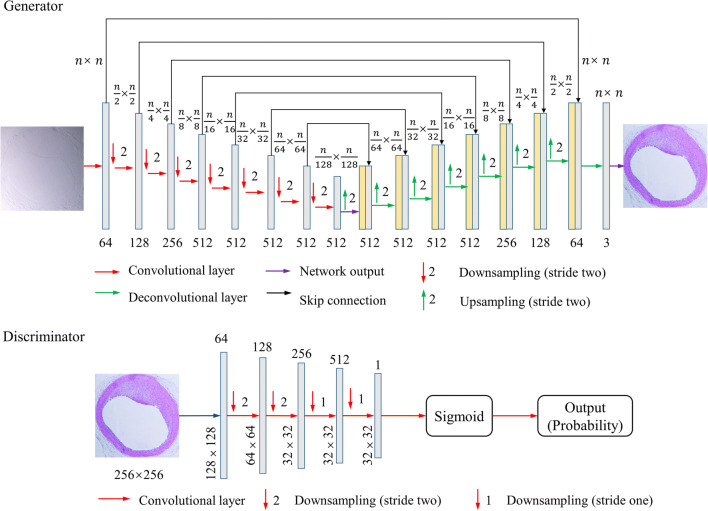
Architecture of virtual staining cGAN. The generator consists of eight convolution layers of stride two that are each followed by a batch-norm module to avoid overfitting of the network. The eight upsampled sections are followed by the deconvolutional layers to increase the number of channels. Each upsampling section contains a deconvolution layer upsampled by stride two. Skip connections are used to share data between layers of the same level. The discriminator is used to discriminate between virtual staining images and histological staining images. It comprises five down blocks, each of which have convolutional layers of stride two to reduce the tensor size. The down block reduces the size of the images while increasing the number of channels to 512 and reduce to 1 followed by a sigmoid activation function. The variable n represents the number of pixels of each image patch that passes through the network.

In this study, we propose a deep learning-based virtual staining method to generate virtually stained images from bright-field microscopic images of unlabeled rat carotid artery tissue sections imaged with a conventional wide-field microscope (Fig. 1). We trained a deep CNN using the concept of cGAN to match the bright-field microscopic images of unstained tissue sections after obtaining standard histological stains (Fig. 2 and Suppl. Fig. 1). Thus, we could replace the histological staining and bright-field imaging steps with the output of the trained neural network, which is fed with the bright-field microscopic images of the unstained tissue.

We demonstrated the applicability of this deep learning-based virtual histological staining method by imaging fresh rat carotid artery tissue samples. The network output created equivalent images that were well matched with the images of the same samples labeled with three different stains—H&E, picrosirius red (PSR), and orcein stain. Furthermore, the staining efficacy of our approach was blindly evaluated by three board-certificated pathologists. They could recognize histopathological features in images generated with our virtual staining technique, and a high degree of agreement was observed between the histologically stained images and the virtually stained images. Quantification of virtual and histological staining in tissue sections shows that our deep learning-based virtual staining method achieved results comparable to the histological staining technique.

## Materials and Methods

### Sample Preparation and Image Acquisition

The carotid artery balloon injury rats (male SD, 200–250 g, purchased from experimental Animal center of Chinese PLA general Hospital) model was built using a previously well-established method [22]. The injured artery was excised aseptically from euthanized animals, washed with phosphate-buffered saline (PBS), and fixed with 4 % buffered formalin for 24 h at 4 °C in the dark. The 4-μm cross sections of paraffin-embedded rat carotid artery were collected and immediately imaged with an inverted microscope (Leica DM IL LED) equipped with a × 10/0.22 NA objective. After a bright-field imaging of unlabeled tissue sections, the corresponding slides were histologically stained with hematoxylin and eosin (H&E), picrosirius red (PSR), and orcein. The images from H&E-, PSR-, and orcein-stained sections were acquired with the same configuration of the microscope. These tissue-staining methods are only performed for the training and validation of the proposed approach. The samples were obtained from the Department of Cardiology, Chinese PLA General Hospital, and were prepared by the Histology Laboratory at Chinese PLA General Hospital. All animal experiments were approved in accordance with the guidelines of the Institutional Animal Care and Use Committee (IACUC) of Chinese PLA General Hospital.

### Preparing Dataset for Training

Our virtual staining networks transformed unstained tissue images to H&E-, PSR-, and orcein-stained images. Following the bright-field imaging, unstained tissue slides underwent a standard histological staining procedure. To acquire the input and target images with the same resolution and field of view (FOV), microscopic imaging of unstained sections and corresponding stained sections was performed at the same imaging system. However, the unstained tissues may be deformed during histological staining. Therefore, it is crucial to register the FOVs of the input-targeted image pairs. In this work, we performed rigid registration to align unstained and stained images. We acquired 60 images for the unstained, H&E-, PSR-, and orcein-stained groups. In total, 240 whole-slide images (WSIs) were obtained. Each WSI (1079 × 1079 pixels) was randomly cropped into 25 smaller overlapping patches (500 × 500 pixels). After eliminating the patches without intima and media, we obtained training image pairs (1800, 1500, and 1500) and testing image pairs (200, 150, and 150) for virtual H&E, PSR, and orcein staining, respectively.

### Conditional Generative Adversarial Network Architecture

To learn the nonlinear mapping from unstained images to standard histological staining of the sample, we utilized a conditional generative adversarial network (cGAN) [23], which is an extension of the generative adversarial network (GAN) [24]. GAN is based on deep convolution learning network, which consists of two networks: generator (G) and discriminator (D). G is responsible for generating new images from prior random data distribution by simulating the real image data distribution. The discriminator D learns a rule to distinguish the images generated by G or the real histological staining images in our case. The two networks have a competitive relationship and are trained at the same time.

In this work, the generator D and the discriminator G of the cGAN comprised a U-net architecture [25] and of PatchGAN [26], respectively, as shown in Fig. 2. The cGAN architecture was updated with the following changes: downsampling path learns image context information and upsampling path target location added to the cGAN model to facilitate input images of 500 × 500 pixels.

To acquire results without generating blurring images, we chose the L1 norm instead of the L2-norm (mean squared error) penalty as a cost function [23, 27]. We defined the loss function of the cGAN as follows:

$$ {L}_{\mathrm{cGAN}}\left(G,D\right)={E}_{x,y\sim {p}_{\mathrm{data}}\left(x,y\right)}\left[\log D\left(x,y\right)\right]+{E}_{x\sim {p}_{\mathrm{data}}(x)}\left[\log \left(1-D\left(x,G(x)\right)\right)\right], $$

where *x* is the input unstained image to the generator, *y* is the ground truth image (the corresponding histological stained image in our case), *z* is a random noise added as dropout in this work, *p*_data_(*x*, *y*) is the joint probability distribution of the training data including pairs of input image *x* and ground truth image *y*, and ${E}_{x,y\sim {p}_{\mathrm{data}}\left(x,y\right)}$ is the expectation of log likelihood of (*x*, *y*). To reduce blurring and generate shaper images, the L1 regularization term was chosen as follows:

$$ {L}_{L1}(G)={E}_{x,y,z}\Big(\left\Vert {\left.y-G\left(x,z\right)\right\Vert}_1\right). $$

The global cost objective of adversarial learning in cGANs is defined as follows:

$$ \mathrm{G}=\arg \underset{G}{\min}\underset{D}{\max }{L}_{\mathrm{cGAN}}\left(G,D\right)+\lambda {L}_{L1}(G). $$

The regularization parameter *λ* is empirically chosen to 100 to balance the adversarial loss and global loss. The convolution kernels of cGAN were set to be 4 × 4. These kernels were randomly initialized using a uniform distribution with a minimum value of 0. We set all biases as random uniform distribution. A dropout rate of 0.5 was used in our experiments. We trained our virtual staining network model for 200 epochs with a learning rate of 0.001 for the generator network and 0.0002 for the discriminator network. Adam optimizer with a batch size of 1 and an exponential decay rate of 0.5 was used in our training. For each iteration of the discriminator, there was one iteration of the generator network.

### Implementation

Our virtual staining network was implemented using Python version 3.6.9. The cGAN was implemented using TensorFlow version 1.12.0. The other Python libraries used were cv2, os, time, tqdm, the Python imaging library (PIL), SciPy, glob, ops, sys, and numpy. We implemented the software on a desktop computer with a Core i7-8700K CPU at 3.20 GHz (Intel) and 12 GB of RAM, running a Linux 4.15.0 operating system. All the experiments including network training and testing were performed on Nvidia GeForce RTX 2080Ti GPU. The other implementation details, including the number of trained patches, the number of epochs, and the training times, are summarized in Suppl. Table 2.

### Evaluation

Calculation of the area of intima, media, and intima-to-media ratio for virtual and standard staining in tissue sections was performed using ImageJ package FIJI (version 1.51) [28]. Data were represented as mean ± SD (standard deviation).

## Results

### Virtual Staining of Rat Carotid Artery Tissue Sections

**Fig. 3.**
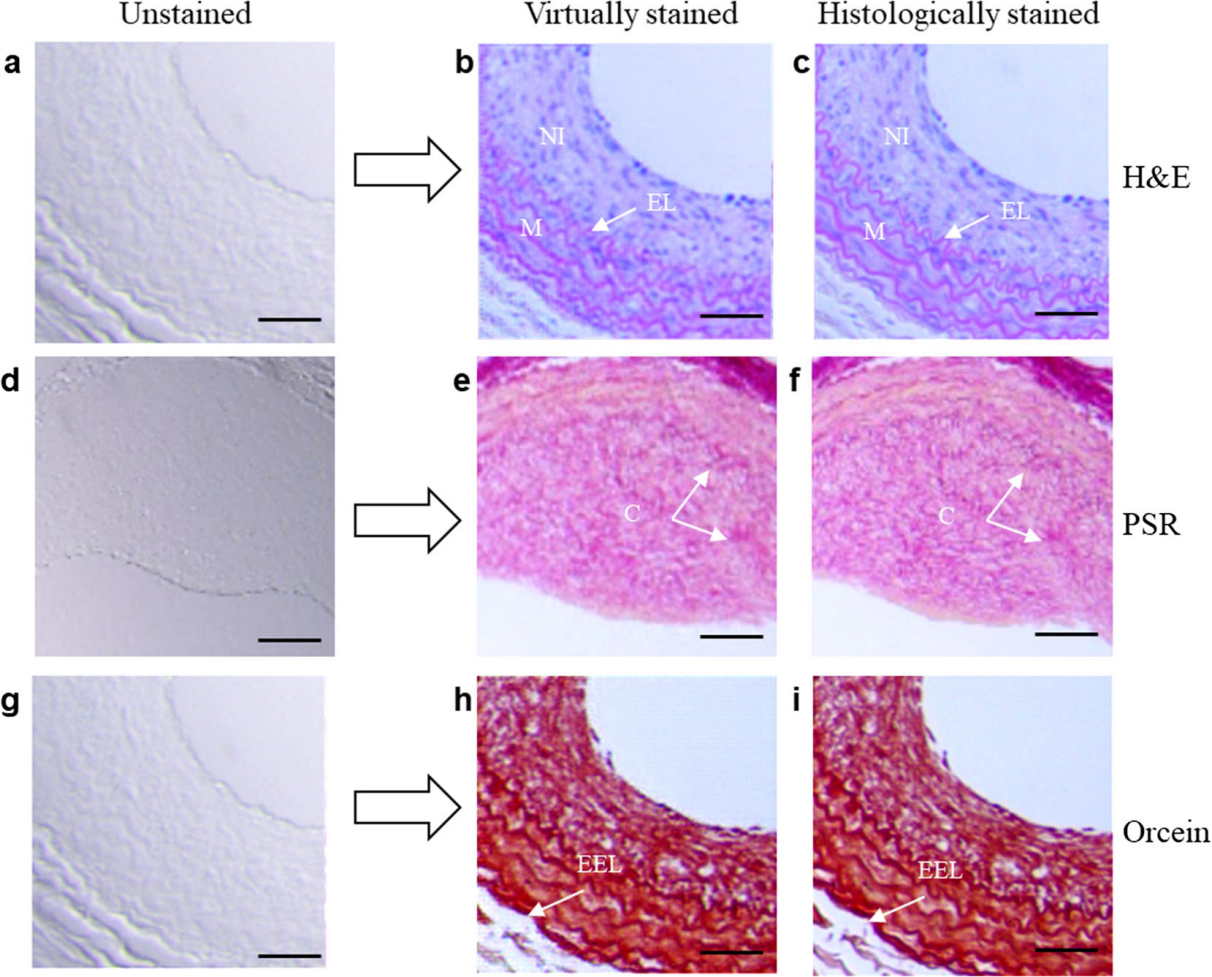
Virtual staining results *versus* the H&E-, PSR-, and orcein-stained images. **a**, **d**, **g** Bright-field images of unstained carotid artery tissue sections used as input of cGAN. **b**, **e**, and **h** Show virtual H&E, PSR, and orcein staining of carotid artery tissues, respectively. **c**, **f,** and **i** show the bright-field images of H&E, PSR, and orcein histologically stained tissues. Note that the neointima (NI), media (M), elastic lamina (EL), collagen (C), and external elastic lamina (EEL) are clearly displayed in both staining techniques. Scale bar, 100 μm.

We present three virtual staining results for carotid artery tissue sections, which were generated by our trained cGANs from the testing dataset, as shown in Fig. 3. These generated images demonstrated that the cGANs can transform bright-field images of unstained tissue sections (Figs. 3a–g) into the corresponding colorized images that are expected from H&E-, PSR-, and orcein-stained tissue sections. Evaluation of Figs. 3b and c shows that the neointima, media, and elastic lamina are present in both H&E staining techniques. Notably, the presence of neointima formation is clearly displayed in both panels. The replication of neointimal cells can also be identified in both virtual staining and histological staining images.

We further trained our cGAN to virtually stain other tissue types using PSR and Orcein stains. Figures 3e and f show the results for virtual staining of an unstained tissue section that matches very well with a bright-field image of PSR staining. These results illustrate that the cGAN can stain patterns of different histological staining for different tissue sections. The virtual PSR staining technique for carotid artery tissue sections in Fig. 3e correctly displays the distribution of collagen. This result is consistent with the histologic appearance in the bright-field images of the same tissue samples after histological staining (Fig. 3f). Similarly, orcein virtual staining of the tissue section shown in Fig. 3h reveals consistently stained external elastic lamina and histological features that correspond to their appearances shown in the bright-field image after histological staining (Fig. 3i).

### Multiple Virtual Stains on the Same Unstained Tissue Section

**Fig. 4.**
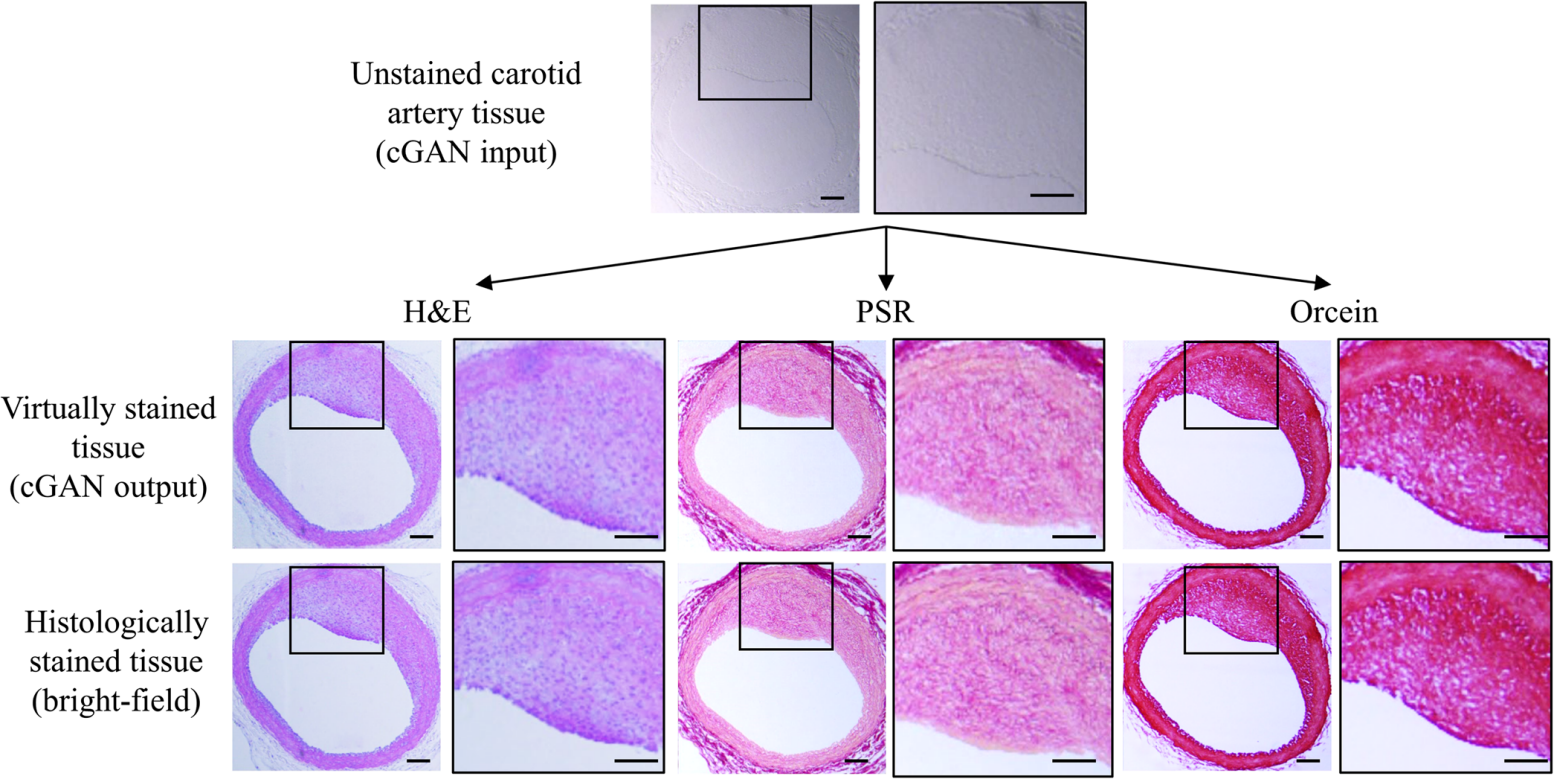
Multiple virtual staining results match the H&E, PSR, and orcein stains for the same unlabeled tissue section. Scale bar, 100 μm.

We trained our deep network to perform multiple virtual stains—H&E stain, PSR stain, and orcein stain—on the same unstained tissue section using StarGAN [29]. This network is capable of learning mappings among multiple stained domains using a single generator. Multiple virtual stains on the same unlabeled tissue section match well with the bright-field images of the three consecutive sections captured after histological staining (Fig. 4). These results illustrate that the StarGAN method can infer multiple types of histologically staining methods from a single bright-field image of an unlabeled sample. In an example of multiple virtual staining, the H&E virtual stain correctly revealed the histological features of the H&E staining and the degree of neointima hyperplasia. Virtual PSR staining captured the histological features of collagen; this is consistent with the histological appearance in the bright-field images captured after histological staining. Similarly, orcein virtual staining revealed consistently stained histological features of elastin in the bright-field images after histological staining.

### Blind Evaluation of Virtual and Histological Staining

To demonstrate the efficacy of our approach, H&E, PSR, and orcein virtual staining and the corresponding histological staining of the same tissue sections were blindly evaluated by three board-certified pathologists. These pathologists were blinded to the staining techniques and asked to apply a grade from 1 to 5 for the quality of the different stains: 5, perfect; 4, very good; 3, good; 2, acceptable; 1, unacceptable. The pathologists could recognize histopathological features, including neointima, media, elastic lamina, collagen, and external elastic lamina, presented in both virtual and histological stains. The blind testing results are summarized in Table 1 (H&E staining). Moreover, the pathologists applied the same score scale (1–5) for PSR and orcein staining features, that is, collagen and external elastic lamina, respectively. The results are summarized in Suppl. Table 1.

**Table 1.** Blind evaluation of virtual and histological H&E staining in carotid artery tissue sections

Tissue number	Pathologist 1			Pathologist 2			Pathologist 3			Average		
	NI	M	EL	NI	M	EL	NI	M	EL	NI	M	EL
1 (VS)	3	3	3	3	3	3	4	4	4	3.33	3.33	3.33
1 (HS)	4	4	3	3	4	4	4	4	4	*3.67*	*4.00*	*3.67*
2 (VS)	3	4	3	3	4	3	4	4	4	3.33	*4.00*	3.33
2 (HS)	4	4	4	3	4	3	4	4	4	*3.67*	*4.00*	*3.67*
3 (VS)	3	3	3	3	4	3	4	5	4	3.33	4.00	3.33
3 (*HS*)	4	4	4	4	4	3	4	5	4	*4.00*	*4.33*	*3.67*
4 (VS)	3	3	3	3	4	3	4	4	4	3.33	*3.67*	3.33
4 (HS)	4	3	4	4	4	3	5	4	4	*4.33*	*3.67*	*3.67*
5 (VS)	3	3	3	3	3	3	4	4	4	3.33	3.33	3.33
5 (HS)	4	4	4	3	4	3	5	5	4	*4.00*	*4.33*	*3.67*

Carotid artery tissue sections were stained with H&E and graded for neointima (NI), media (M), and elastic lamina (EL). *HS,* histologically staining; *VS*, virtually staining. The winner (and tied) average scores are in italics

Furthermore, the three pathologists were asked to calculate the intima thickness (IT), intima area (IA), media area (MA), and intima-to-media ratio (IMR) to evaluate the neointima hyperplasia in both virtual and histological stains. The IMR was calculated as the mean area of intima divided by the mean area of media. We compared the relative error (RE) between the histologically stained and virtually stained samples to evaluate the quantification accuracy. The quantification results are summarized in Table 2 (H&E staining).

**Table 2.** Quantification of virtual and histological H&E staining in carotid artery tissue sections

Tissue number	IT (μm)	RE of IT (%)	IA (μm^2^)	RE of IA (%)	MA(μm^2^)	RE of MA (%)	IMR
1 (VS)	228.5 ± 0.9	0.2	261,474.5 ± 2253.0	0.8	175,412.1 ± 7082.3	3.3	1.49
1 (HS)	228.1 ± 1.9		263,530.1 ± 1136.2		169,753.7 ± 3665.8		1.55
2 (VS)	244.6 ± 1.3	1.6	128,655.6 ± 5884.7	4.1	149,911.9 ± 7493.7	0.1	0.86
2 (HS)	248.5 ± 1.9		134,151.7 ± 1535.5		150,026.1 ± 2436.2		0.89
3 (VS)	206.9 ± 2.9	0.1	259,228.6 ± 15,163.9	0.3	183,164.8 ± 14,245.2	4.1	1.42
3 (HS)	206.6 ± 1.1		258,425.0 ± 14,351.3		175,953.6 ± 7619.6		1.47
4 (VS)	140.0 ± 1.4	1.6	254,563.0 ± 12,650.4	3.6	205,179.6 ± 10,924.2	12.7	1.24
4 (HS)	137.9 ± 2.0		264,064.6 ± 8300.7		181,998.4 ± 5369.6		1.45
5 (VS)	223.6 ± 2.5	1.1	128,244.5 ± 1436.1	5.6	162,862.3 ± 4462.2	1.2	0.79
5 (HS)	226.1 ± 0.7		135,838.3 ± 414.6		164,876.6 ± 1722.3		0.82

*IT*, intima thickness; *IA*, intima area; *MA*, media area; *IMR,* intima-to-media ratio. *RE*, relative error. *HS*, histologically staining; *VS*, virtually staining

Our results indicate that the pathologists could recognize neointima hyperplasia-related histopathological features using both staining techniques and with a high degree of agreement between the techniques.

## Discussion

Histological staining analysis is performed as a “golden standard” in diagnostic pathology. It is widely used to identify carotid artery tissue constituents such as intima, media, collagen, and elastic lamina, under light microscope with a high magnification objective. However, the histological staining process is laborious, and image quality of tissue staining images is variable because of the different staining protocols, imaging devices, and human-to-human variations. Such staining processes lead to a barrier in developing standard and fast histological image analysis systems. Notably, the standard histological staining procedure can only be performed on single tissue sections with one type of stain. Therefore, the identification of various components of the same tissue section is highly required.

In this work, we proposed a trained cGAN that virtually stains the label-free fresh tissue sections into the corresponding H&E-, PSR-, and orcein-stained sections of rat carotid artery tissue samples. This deep learning-based method can provide a digital staining of label-free tissue sections, which can bypass the lengthy and laborious tissue preparation process and can mitigate human-to-human variations for standard histological staining of tissue samples. We trained this statistical learning-based network using white light images of unstained sections to learn from histologically stained tissue sections. A blind test was performed to demonstrate the performance of the network by board-certified pathologists. After training, this virtual staining method can be enhanced by combing the unstained images acquired using other advanced label-free imaging techniques, for example, autofluorescence microscopy and Raman microscopy.

For the training process, it is important to match the bright-field images of unstained sections and the corresponding images acquired after histological staining. However, during the whole histological staining process, tissue can be deformed or contaminated by dust, resulting in challenges of the loss function in the training step. To reduce these impacts on the performance of the network, we performed a global co-registration using rigid algorithm (https://www.mathworks.com/help/images/registering-multimodal-mri-images.html). We further performed a visual inspection of the registered unstained and histological staining image pairs to eliminate the images that included dust or large deformations.

It is important to note that, gradient descent-based GAN training is not always locally convergent partly due to the discontinuous dataset, such as bright-filed microscopic images of unstained tissue sections that used in this study. Here, we transfer bright-field images of unlabeled carotid artery tissue into equivalent images of histologically stained versions of the same samples using conditional generative adversarial network, which are extensions of GANs where both generator and discriminator are conditioned on additional information. To stable the training, we chose L1 regularization term to reduce blurring and generate shaper images. In addition, the regularization parameter *λ* was used for balancing the adversarial loss and global loss.

Our virtual staining procedure is implemented by training separate cGAN for each staining. To reveal multiple virtual stains, namely H&E, PSR, and orecin stains in our case, we trained the StarGAN network to generate three different stained images from the same unstained image. This is practical for histological applications, that is, the number of staining types on the same unlabeled tissue will be unlimited. This virtual staining method can provide fast pathological diagnosis of various components. In this study, it can generate three different type of stains, including H&E, PSR, and orcein, within 3 s with current hardware configuration. Although this is an improvement compared to the virtual stain cGAN for single tissue stain combinations, the multiple virtual stain image is a little inferior for generating high-quality multiple virtual staining images. Within this network, a generator generates images of multiple domains, solving the problem of image pairing and reducing network parameters. The output results demonstrated that multiple virtual staining images generated from an unstained image simultaneously can express the main staining components according to the stained images.

Our study has some limitations. Firstly, the output image of network needs to be corrected according to the stained images. Substantially, a wide-scale, randomized evaluation of virtual staining images by more pathologists will regulate the network to achieve a high-quality image. This is very useful for fast analysis of bright-field microscopic images of tissue sections in the future. In addition, the combination of high-resolution bright-field images of unlabeled tissue sections with virtual staining in the deep learning-based network results in high-resolution virtual staining images with more detail in histological features. Finally, the quantitative virtual staining evaluation indexes, including the quantitative image-based metrics like SSIM, are required to assist pathologists during routine clinical diagnosis. Notably, this virtual staining method could be transferred into a clinical research on unlabeled human artery tissue samples, which would be necessary to improve the diagnostic efficiency and accuracy of the trained network with the histological stains.

## Conclusions

In conclusion, we have developed a deep learning-based virtual staining method that transformed bright-field microscopic images of unlabeled tissue sections into their corresponding images of histological staining of the same samples using a conditional generative adversarial network model. This virtual staining method has been validated by pathologists *via* blind evaluation. Further improvement of this method will be focused on the combination of advanced other label-free microscopic imaging modalities and the evaluation by large-scale randomized clinical study. We envision that this virtual staining method will provide strong support to the applications of histology analysis in study for CAD.

## Electronic supplementary material

ESM 1 (DOCX 349 kb)

## Data Availability

The dataset and source code of this study are available from the corresponding authors upon request.
