# Supplementary material for: Deep Learning for Virtual Histological Staining of Bright-Field Microscopic Images of Unlabeled Carotid Artery Tissue
Source: Mol Imaging Biol. 2020 Jun 8;22(5):1301–9. doi: 10.1007/s11307-020-01508-6 (PMC7497459; doi:10.1007/s11307-020-01508-6)
Supplement: Supplementary file 1 — (DOCX 349 kb) [file 11307_2020_1508_MOESM1_ESM.docx]

Supplementary material

*Article name:* Deep learning for virtual histological staining of bright-field microscopic images of unlabeled carotid artery tissue

Journal name: Molecular imaging and biology

Dan Li^1,2,+^, PhD, Hui Hui^2,3,+^, PhD, Yingqian Zhang^4,+^, MD, Wei Tong^4^, MD, Feng Tian^4^, MD, Xin Yang^2^, PhD, Jie Liu^1,*^, PhD, Yundai Chen^4,*^, MD, Jie Tian^2,3,5*^, PhD

^1^Department of Biomedical Engineering, School of Computer and Information Technology, Beijing Jiaotong University, Beijing 100044, China

^2^CAS Key Laboratory of Molecular Imaging, Institute of Automation, Chinese Academy of Sciences, Institute of Automation, Beijing 100190, China

^3^University of Chinese Academy of Sciences, Beijing, China

^4^Department of Cardiology, Chinese PLA General Hospital, Beijing, 100853, China

^5^Beijing Advanced Innovation Center for Big Data-Based Precision Medicine, School of Medicine, Beihang University, Beijing, 100083, China

**Correspondence to:* [jieliu@bjtu.edu.cn](mailto:jieliu@bjtu.edu.cn) (Jie Liu), [cyundai@vip.163.com](mailto:cyundai@vip.163.com) (Yundai Chen), [jie.tian@ia.ac.cn](mailto:jie.tian@ia.ac.cn) (Jie Tian)

Supplemental Tables

**Supplemental Table 1. Blind evaluation of virtual and histological PSR and Orcein staining in carotid artery tissue sections**

| Tissue number | Pathologist 1 | | | Pathologist 2 | | | Pathologist 3 | | | Average | | |
| --- | --- | --- | --- | --- | --- | --- | --- | --- | --- | --- | --- | --- |
|  | NI | C | EEL | NI | C | EEL | NI | C | EEL | NI | C | EEL |
| 1 (VS) | 3 | 3 | 3 | 3 | 3 | 3 | 4 | 4 | 4 | 3.33 | 3.33 | 3.33 |
| 1 (HS) | 4 | 4 | 4 | 3 | 3 | 4 | 4 | 4 | 4 | **3.67** | **3.67** | **4.00** |
| 2 (VS) | 3 | 4 | 3 | 3 | 4 | 3 | 4 | 4 | 4 | **3.33** | 4.00 | 3.33 |
| 2 (HS) | 3 | 4 | 4 | 3 | 4 | 4 | 4 | 5 | 4 | **3.33** | **4.33** | **4.00** |
| 3 (VS) | 3 | 3 | 4 | 3 | 4 | 3 | 4 | 4 | 4 | 3.33 | **3.67** | **3.67** |
| 3 (HS) | 4 | 3 | 3 | 4 | 4 | 3 | 5 | 4 | 4 | **4.33** | **3.67** | **3.33** |
| 4 (VS) | 3 | 3 | 3 | 3 | 4 | 4 | 4 | 4 | 4 | 3.33 | 3.67 | 3.67 |
| 4 (HS) | 4 | 4 | 3 | 4 | 4 | 4 | 5 | 4 | 4 | **4.33** | **4.00** | 3.67 |
| 5 (VS) | 3 | 3 | 3 | 3 | 4 | 3 | 4 | 3 | 4 | 3.33 | 3.33 | 3.33 |
| 5 (HS) | 4 | 4 | 4 | 3 | 4 | 4 | 5 | 4 | 4 | **4.00** | **4.00** | **4.00** |

Carotid artery tissue sections were stained with PSR and Orcein and graded for neointima (NI), collagen (C) and external elastic lamina (EEL). 5, perfect; 4, very good; 3, good; 2, acceptable; 1, unacceptable. HS, histologically staining; VS, virtually staining. The winner (and tied) average scores are in bold.

**Supplemental Table 2. Network training details for different stains.**

| Virtual staining network | # of training patches | # of epochs | Training time (hours) |
| --- | --- | --- | --- |
| H&E | 1800 | 200 | 12.857 |
| PSR | 1500 | 200 | 10.714 |
| Orcein | 1500 | 200 | 10.714 |

Supplemental Figure:


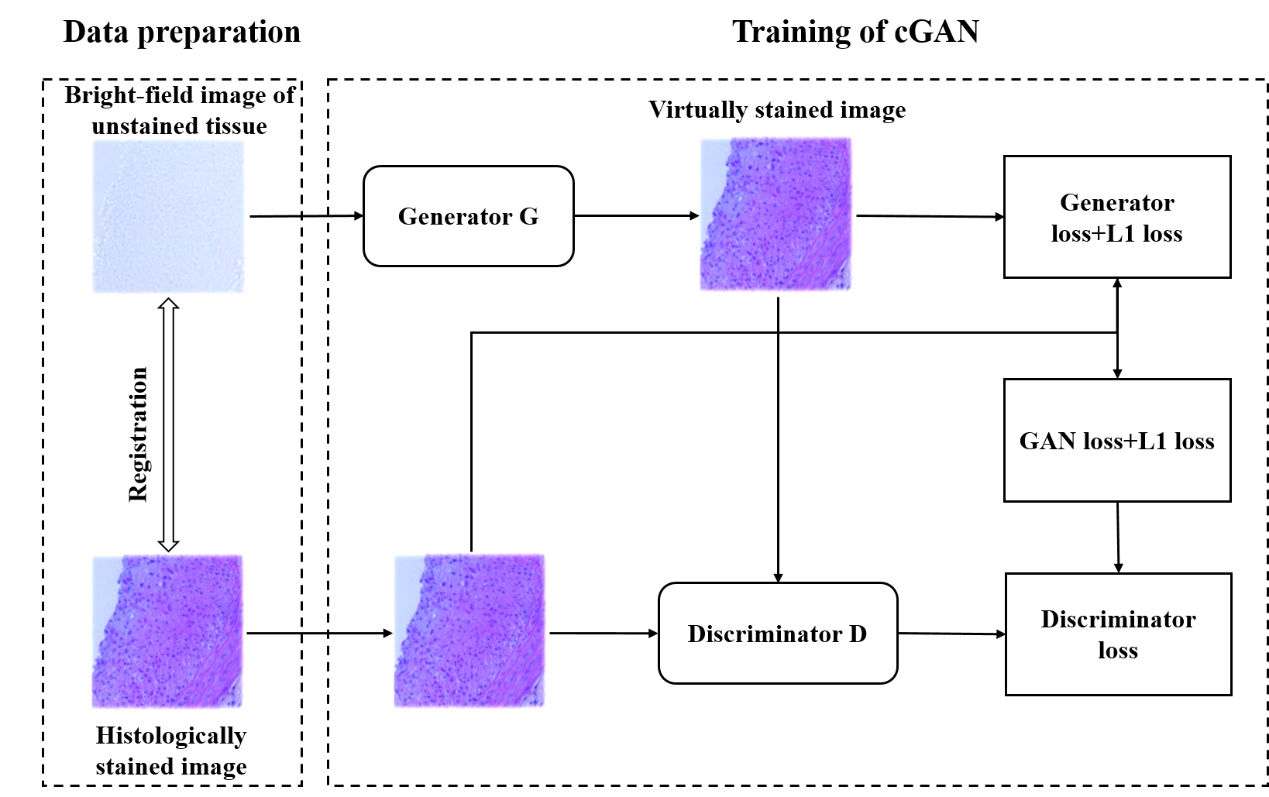


Supplemental Figure 1. Training process of the virtual staining neural network using a cGAN.
